# Bridging the bench‐to‐bedside divide in microbiome research

**DOI:** 10.1002/ctm2.70358

**Published:** 2025-05-29

**Authors:** Sondra Turjeman, Omry Koren

**Affiliations:** ^1^ Azrieli Faculty of Medicine Bar‐Ilan University Safed Israel

## 1 INTRODUCTION

Microbiome research has expanded rapidly in recent years, producing a large volume of publications across many clinical fields. However, despite the numerous studies reporting correlations between microbial dysbiosis and host health and disease states,^1^, ^2^ few findings have translated into interventions that impact clinical care. For many healthcare professionals, this gap between discovery and application has become a clear call to action, underscoring the need for new translational strategies that bridge basic science and clinical relevance.^3^

**FIGURE 1:**
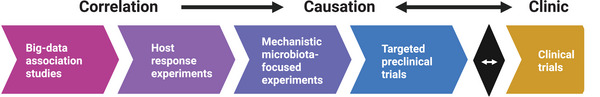
Iterative research model for clinical translation of microbiota research. Many studies begin with large human cohorts and extensive datasets (from left, pink), which serve as the basis for generating a working hypothesis. These include disease cohorts, population cohorts, digital health records, omics data and other deep phenotyping. The next step typically involves host‐focused proof‐of‐concept studies, such as faecal microbiota transplantation (FMT) from, e.g., diabetic individuals into germ‐free mice, to determine whether the phenotype can be replicated. Cell lines, organoids, organs‐on‐a‐chip and animal models can all be used in this stage. Once causality is established, researchers should investigate the underlying mechanisms using targeted models – for instance, monocolonisation with candidate microbes and detailed cellular phenotyping under controlled conditions, in vitro set‐ups, labelling metabolism experiments in vitro and in vivo, and bioreactor culturing. Once mechanistic insights are achieved, interventions can be designed and tested in preclinical models. Interventions can include prebiotics, probiotics, or postbiotics that restore key microbial metabolites implicated in pathways or FMT. If these interventions show safety and efficacy, clinical trials would follow. Should trial outcomes fall short of preclinical results, iterative testing, such as adjusting dosage or exploring co‐administration strategies (e.g., synbiotics), may be required.

In a recent *Cell* perspective,^4^ we and our co‐authors proposed a structured, iterative approach to improve microbiome translation from early discovery studies through to clinical trials (Figure 1). While this framework emphasises experimental models and data integration, its success ultimately depends on multidisciplinary collaboration between clinicians and researchers. Broader progress in microbiome translation will depend on better integration of clinical insight with experimental design; identifying meaningful cross‐species phenotypes, defining clinically relevant endpoints, and co‐developing translational models will be key to making microbiome science more clinically actionable.

## 2 FROM CLINICAL PATTERNS TO DATA‐DRIVEN HYPOTHESES

Many research questions in microbiome science begin with clinical observations of variability in patient response, symptom clustering, or disease trajectories that do not follow expected patterns. When these insights are systematically recorded and paired with biological sampling, they become a foundation for hypothesis generation.

Specifically, the growing availability of large, deeply phenotyped cohorts allows for exploration of clinical questions at scale. By combining rich clinical metadata with microbiome and metabolome profiling, researchers can build large, diverse databases, so‐called ‘meta‐cohorts’, which can be leveraged to reveal robust and reproducible associations between a variety of host states and multi‐omics profiles.^5^ Statistical modelling and machine learning approaches can then be used to identify conserved microbial signatures, host–microbe interactions or functional pathways associated with specific clinical phenotypes^6^ which can then be examined mechanistically to better understand disease aetiology and define biomarkers for diagnosis or therapeutic intervention.

## 3 FROM HYPOTHESES TO MECHANISMS: EXPERIMENTAL VALIDATION

Once robust associations are identified through clinical observations and large‐scale data analysis, the next step is to determine whether these patterns reflect causal relationships. Experimental models, ranging from in vitro gut culture systems^7^ to gnotobiotic animals^8^, allow researchers to examine how specific microbial strains, functions, or metabolites influence host physiology or disease progression. Proof‐of‐concept studies often begin with FMT from patient subgroups into germ‐free or antibiotic‐treated mice. If a clinical phenotype, such as altered glucose tolerance,^9^ behaviour,^10^ or treatment responsiveness,^11^ is transferred, it suggests that the microbiome may be mechanistically involved in the host state. These findings can then be further dissected using reductionist models (Figure 1), such as monocolonisation in germ‐free animals, microbiota–organoid systems, or in vitro and ex vivo co‐culture assays, to pinpoint the specific microbes, metabolites and host pathways driving the observed effects. One example of an iterative study that confirms causative effects and dives into mechanistic understandings is the work by Fluhr et al., which moved from population‐level associations to mouse models, identified a microbiota‐derived metabolite that modulates weight gain, and then returned first to mice and then to humans to confirm the signal, demonstrating a full translational loop.^12^ Importantly, the more closely preclinical models capture human physiology and clinical heterogeneity, the greater their potential to yield findings that are translatable to patient care.

## 4 WHY TRANSLATION FAILS: LIMITATIONS OF PRECLINICAL MODELS

Despite careful experimental design, many findings from microbiome interventions do not replicate in human studies. One example is the use of FMT for improving metabolic health. In mouse models, FMT from lean and obese donors transfers the respective donor phenotypes to colonised mice, and co‐housing between lean‐colonised and obese‐colonised mice was found to offer protective effects against obesity due to coprophagy.^13^ In contrast, in clinical trials, similar interventions have shown far more modest results. In a double‐blind, placebo‐controlled trial in individuals with severe obesity and metabolic syndrome, FMT from lean donors led to transient improvements in insulin sensitivity, but only when coupled with low‐fermentable fibre supplements, and no effects on body weight were observed.^14^

A key reason for these discrepancies lies in the fundamental physiological, immunological, and ecological differences between animal models – especially mice – and humans. These include differences in gut anatomy, diet, microbiota composition and density, immune system development, and pharmacokinetics (see Figure 2 of Turjeman et al.^4^), which can significantly alter the cross‐species translatability of microbially focused or derived interventions.^15^, ^16^ Rather than discounting translational failures, they should inform a more nuanced approach to translation. Aligning model design more closely with human biology and designing clinical trials that are responsive to preclinical insights – and vice versa – is essential for moving the field forward.

## 5 THE ROAD AHEAD: SUPPORTING MORE TRANSLATABLE MICROBIOME SCIENCE

As the field matures, the next phase of microbiome research requires greater emphasis on mechanisms and clinical relevance. Emerging strategies – including the development of defined microbial consortia,^17^ engineered probiotics^18^ and various metabolite‐based therapies – aim to move beyond broad‐spectrum interventions toward targeted, mechanistically informed approaches. These tools hold promise for increasing reproducibility and improving regulatory pathways.

Yet their success will depend on more than molecular innovation. Trial design must be adapted to the complexities of microbiome interventions, accounting for factors such as baseline microbial composition, host diet and inter‐individual variability. Human‐relevant model systems, including wilded mice,^19^ humanised gnotobiotic models,^20^ and personalised, host‐derived organoid platforms, may help bridge some of the translational gaps identified in earlier studies.

Throughout this process, clinician involvement remains essential, not only in hypothesis generation and trial design, but also in defining meaningful outcomes and guiding real‐world application. As new tools and data sources emerge, so too will opportunities to make microbiota‐focused diagnostics and therapeutics a more routine part of clinical care. Realising this potential will require a shift from parallel research tracks to an integrated, iterative approach where clinical insight and experimental discovery move forward together.
